# A Predictive Model for Acute Kidney Injury Based on Leukocyte-Related Indicators in Hepatocellular Carcinoma Patients Admitted to the Intensive Care Unit

**DOI:** 10.1155/mi/7110012

**Published:** 2025-04-16

**Authors:** Xiulan Peng, Yahong Cai, Huan Huang, Haifeng Fu, Wei Wu, Lifeng Hong

**Affiliations:** ^1^Department of Oncology, The Second Affiliated Hospital of Jianghan University, Wuhan 430050, Hubei Province, China; ^2^Department of Oncology, Suizhou Zengdu Hospital, Suizhou 441300, Hubei, China; ^3^Department of Hepatopancreatobiliary Surgery, Sinopharm Dongfeng General Hospital, Hubei University of Medicine, Shiyan 442008, Hubei, China; ^4^Department of Critical Care Medicine, Renmin Hospital of Wuhan University, Wuhan 430000, Hubei Province, China; ^5^Department of Cardiology, The Second Affiliated Hospital of Jianghan University, Wuhan 430050, Hubei Province, China

**Keywords:** acute kidney injury, hepatocellular carcinoma, intensive care unit, mortality, white blood cell

## Abstract

**Background:** This study aimed to develop and validate a straightforward clinical risk model utilizing white blood cell (WBC) counts to predict acute kidney injury (AKI) in critically sick patients with hepatocellular carcinoma (HCC).

**Methods:** Data were taken from the Medical Information Mart for Intensive Care-IV (MIMIC-IV) database for the training cohort. Data for an internal validation cohort were obtained from the eICU Collaborative Research Database (eICU-CRD), while patients from our hospital were utilized for external validation. A risk model was created utilizing significant indicators identified through multivariate logistic regression, following logistic regression analysis to determine the primary predictors of WBC-related biomarkers for AKI prediction. The Kaplan–Meier curve was employed to evaluate the prognostic efficacy of the new risk model.

**Results:** A total of 1628 critically sick HCC patients were enrolled. Among these, 23 (23.2%) patients at our hospital, 84 (17.9%) patients in the eICU-CRD database, and 379 (35.8%) patients in the MIMIC-IV database developed AKI. A unique risk model was developed based on leukocyte-related indicators following the multivariate logistic regression analysis, incorporating white blood cell to neutrophil ratio (WNR), white blood cell to monocyte ratio (WMR), white blood cell to hemoglobin ratio (WHR), and platelet to lymphocyte ratio (PLR). This risk model exhibited robust predictive capability for AKI, in-hospital mortality, and ICU mortality across the training set, internal validation set, and external validation set.

**Conclusion:** This risk model seems to have practical consequences as an innovative and accessible tool for forecasting the prognosis of critically ill HCC patients, which may, to some degree, aid in identifying equitable risk assessments and treatment strategies.

## 1. Introduction

Acute kidney injury (AKI) frequently occurs in hospitalized individuals. The intensive care unit (ICU) exhibited a higher rate of AKI compared to the general population. Mandelbaum et al. [1] reported that stage I AKI occurred in 57% of critically sick patients, with a death incidence of 13.9% among those who developed AKI, compared to 6.2% in those who did not. Nisula et al. [2] reported that 17 Finnish ICUs had a 39.3% incidence of AKI. Individuals with AKI incur care costs around ten times more, alongside an elevated chance of developing chronic kidney disease (CKD) and heightened morbidity and mortality rates [3–5].

Hepatocellular carcinoma (HCC), a highly prevalent cancer, constitutes 8.2% of global cancer-related deaths [6]. A prior study indicated that individuals with HCC often experienced AKI. A substantial cohort study of a Danish population revealed that 33% of HCC patients experienced AKI within 1 year [7]. Moreover, patients with HCC who experienced AKI exhibited a poorer prognosis compared to those without AKI. Following hepatectomy for HCC, Lim et al. [8] found that patients with AKI exhibited significantly elevated mortality and major morbidity rates (37% and 69%) compared to those without AKI (6% and 22%). Consequently, the identification of biomarkers for the early detection of AKI is crucial for the prompt provision of suitable clinical care for HCC patients in ICU environments.

AKI prediction is a pressing concern for critically ill patients. AKI has been associated with many biomarkers, such as neutrophil gelatinase-associated lipocalin (NGAL), cystatin C, and kidney injury molecule-1 (KIM-1) [9]. However, due to their high cost, acquiring these predictors proved challenging. Some severity scores have been utilized in predicting AKI. Nonetheless, these scoring methods exhibit inadequate specificity and sensitivity and have not been implemented in clinical environments [10]. Consequently, a risk model that is easily accessible and demonstrates robust predictive ability is essential for AKI prediction in critically sick patients.

Numerous researchers have determined that predicting AKI in ICU environments using markers related to white blood cells (WBCs) is straightforward. The preoperative systemic immune-inflammation index (SII) has been identified as a unique, independent predictor of postoperative AKI in patients with HCC [11]. The predictive capabilities of the WBC-related indicators-based risk model for AKI prediction in critically ill HCC patients remain unexamined. This study aimed to develop an AKI prediction model for HCC patients in ICU environments by using leukocyte-related indicators.

## 2. Methods

### 2.1. Data Source

The data of this study were extracted from the Medical Information Mart for Intensive Care IV database (MIMIC-IV) [12], the eICU Collaborative Research Database (eICU-CRD) [13] and the Renmin Hospital of Wuhan University. The Declaration of Helsinki was followed when conducting the study. Due to the data used in this study were extracted from public databases, it was exempt from the requirement for informed consent from patients and approval of the ethics review committee. We were given authorization to take information out of the eICU-CRD and MIMIC-IV databases [14].

### 2.2. Study Population

**Figure 1:**
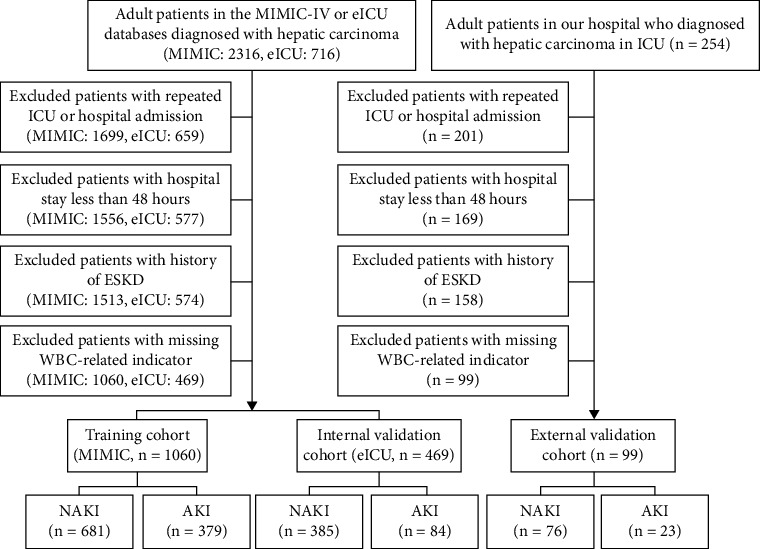
The flow chart of this study.

We extracted adult HCC patients from the eICU-CRD database, MIMIC-IV, and the ICU of Renmin Hospital of Wuhan University using ICD-9 and ICD-10 diagnosis codes, covering the period from January 2021 to July 2022. AKI was diagnosed according to KDIGO-AKI criteria based on serum creatinine levels within the initial 48 h after ICU admission [15]. Patients deemed ineligible possess one of the following conditions: Patients with less than 48 h in the hospital, recurrent ICU or hospital admissions, end-stage renal disease (ESRD), and those with absent values for WBC-related indicators had all missing values for WBC-related indicators completely deleted. The eICU-CRD database provided the internal validation cohort, whilst the MIMIC-IV database constituted the training cohort. Furthermore, data obtained from our hospital served as external validation (Figure 1).

### 2.3. Clinic Variables and Outcomes

The retrieved variables include demographics, vital signs, laboratory tests, scoring systems, complications, comorbidities, and drug usage. All data were gathered within 24 h of ICU admission. The specific variables were presented in Table 1. The principal outcome was AKI, determined according to the KDIGO guidelines for serum creatinine levels within 48 h. The secondary outcomes encompassed the severity of AKI, duration from ICU admission to new AKI onset, persistence of AKI, progression of AKI, utilization of continuous renal replacement therapy (CRRT), administration of vasopressors, application of mechanical ventilation, use of diuretics, incidence of acute heart failure, acute respiratory failure, acute hepatic failure, sepsis, length of ICU stay, length of hospital stay, in-hospital mortality, and ICU mortality. AKI progression, AKI recovery, and persistent AKI were identified according to the prior findings [16]. New AKI was identified in patients who did not exhibit AKI within the initial 48 h of ICU admission but subsequently acquired AKI during their continued ICU stay.

### 2.4. Statistical Analysis

All analyses were conducted using R (version 4.1.0). A statistically significant difference was established as *p*  < 0.05. A novel AKI prediction risk model (risk score=1.22 × WNR+1.03 × lg(WMR)+0.97 × WHR+0.87 × lg(PLR)) was derived from the outcomes of a multivariate logistic regression analysis. The optimal cutoff value for the novel risk model was determined utilizing the receiver operating characteristic curve (ROC). Based on the optimal cutoff value, the high-risk and low-risk groups were established. The clinical utility of the risk model was evaluated by decision curve analysis (DCA). Furthermore, univariate and multivariate logistic regressions were conducted to assess the risk model for predicting diverse clinical outcomes. The forest plot displayed subgroup analysis. Waterfall plots were employed to demonstrate the relationship between risk scores and clinical outcomes [17]. We adhered to the methodologies outlined by Huang et al. [18] and Cai et al. [19].

## 3. Results

### 3.1. Characteristics

Finally, 1628 critically ill HCC patients were enrolled. AKI occurred in 23 (23.2%) patients in our hospital, 379 (35.8%) patients in the MIMIC-IV database, and 84 (17.9%) patients in the eICU-CRD database. Patients were divided into two groups: those with and those without AKI. The baseline characteristics were depicted in Table 1 and Supporting Information Table S1.

### 3.2. A Novel Risk Model Was Established Based on White Blood Cell-Related Indicators

The logistic regression analysis was performed to identify the potential predictors for AKI and the WNR, WMR, WHR, and platelet to lymphocyte ratio (PLR) were included (Table 2), hence, the prediction risk model was as follows: 1.22 × WNR+1.03 × lg(WMR)+0.97 × WHR+0.87 × lg(PLR).

### 3.3. The Novel Model's Performance in Acute Kidney Injury Prediction

**Figure 2:**
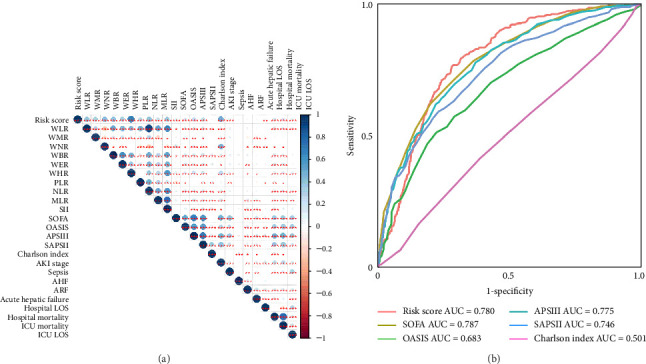
Clinical correlation and predictive ability of serum inflammatory biomarkers in the training set. (A) The correlations between risk score with other serum inflammatory biomarkers, severity score, and clinical outcomes. (B) ROC curves of risk score and severity score for the prediction of acute kidney injury among critically ill hepatocellular carcinoma patients.

Figure 2A demonstrates a substantial correlation between the risk score and WBC-related markers, severity scores, and clinical outcomes in the training set. The findings indicated that the WBC-related risk model (AUC: 0.780) exhibited a robust predictive capability for AKI in comparison to the SOFA score (AUC: 0.787) and APSIII score (AUC: 0.775). Furthermore, our risk model demonstrated superior AKI prediction ability compared to the OASIS score (AUC: 0.683, Figure 2B), SAPSII score (AUC: 0.746, Figure 2B), and Charlson index (AUC: 0.501, Figure 2B) within the training set. ROC analysis was used to ascertain the best cut-off value for the WBC-related indicators-based risk model, then categorizing participants into low-risk (≤ 6.37) and high-risk (> 6.37) groups based on this value.  Table 3 demonstrated the variation in serum creatinine levels prior to and subsequent to the diagnosis of AKI. The findings indicated that the high-risk group exhibited elevated serum creatinine levels at ICU admission, at the initial AKI diagnosis, at 48 h post-diagnosis, and showed positive alterations in serum creatinine within the training set. In the internal validation set, the high-risk group had elevated serum creatinine levels at ICU admission, at the first AKI diagnosis, and 48 h post-AKI diagnosis in comparison to the low-risk group (*p*  < 0.05, Table 3).

Table 4 illustrates that, in comparison to the low-risk group, the high-risk group exhibited a greater proportion of patients with AKI, varying severity of AKI, new instances of AKI, persistent AKI, and progression of AKI, alongside a higher incidence of patients requiring continuous renal replacement therapy (CRRT), vasopressors, mechanical ventilation, acute respiratory failure, acute hepatic failure, sepsis, in-hospital mortality, and mortality in the intensive care unit (ICU). Moreover, the high-risk group exhibited an extended duration between ICU admission and AKI, as well as prolonged ICU and hospital stays in the training set (*p*  < 0.05, Table 4). The internal and external validation sets yielded comparable results (*p*  < 0.05, Supporting Information Table S2).

**Figure 3:**
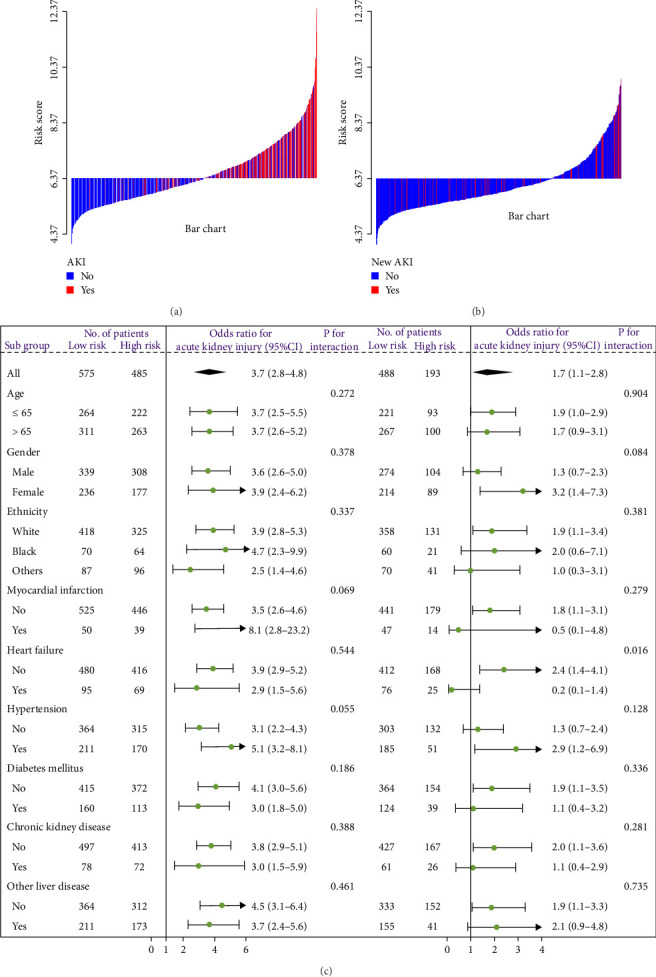
The waterfall plots and forest plots of the high-risk group and low-risk group for the prediction of acute kidney injury (AKI) and new AKI among critically ill hepatocellular carcinoma (HCC) patients in the training set. The waterfall plot of risk score for each patient of (A) AKI and (B) new AKI. (C) The subgroup analysis of the risk score in individuals with critically ill HCC patients for AKI and new AKI.

**Figure 4:**
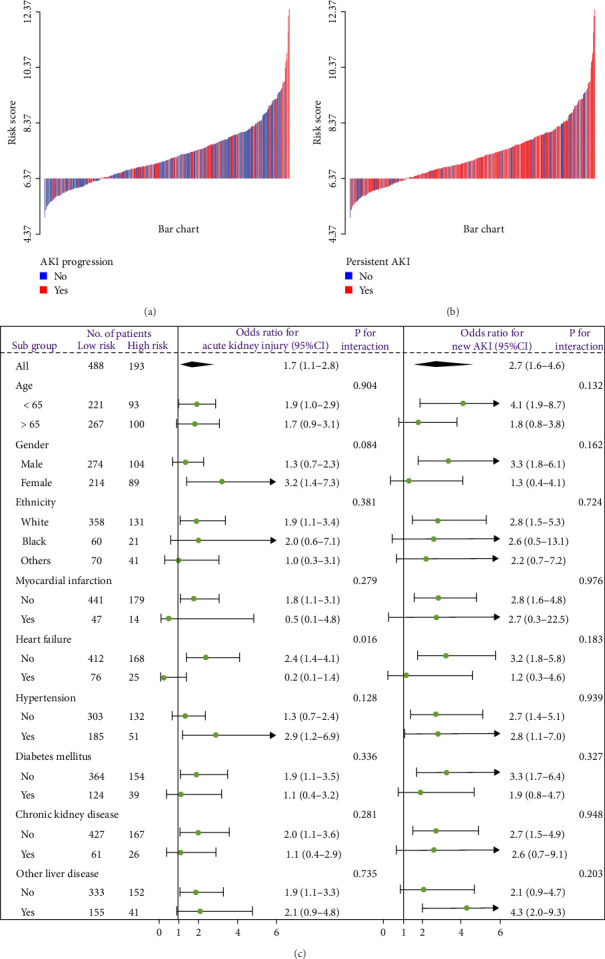
The waterfall plots and forest plots of the high-risk group and low-risk group for the prediction of persistent acute kidney injury (pAKI) and AKI progression among critically ill hepatocellular carcinoma patients in the training set. The waterfall plot of risk score for each patient of (A) pAKI and (B) AKI progression. (C) The subgroup analysis of the risk score in individuals with AKI for pAKI and AKI progression.

A waterfall plot was employed to assess the relationship between the risk score and the clinical outcome. A higher risk score correlates with an increased incidence of AKI, the emergence of new AKI cases, development of AKI, and a greater prevalence of persistent AKI, as demonstrated in Figures 3 and 4. Moreover, in nearly all subgroups within the training set of the predetermined subgroup analysis, patients classified as high risk exhibited a greater likelihood of AKI, new-onset AKI, progression of AKI, and persistent AKI compared to those in the low-risk category (Figures 3C, and 4C). The internal validation set yielded comparable results (Supporting Information Figure S1). The high-risk cohort of critically sick HCC patients had an elevated risk of AKI, new-onset AKI, persistent AKI, and progression of AKI. The crude odds ratios (ORs) for AKI, new AKI, persistent AKI, and AKI progression were 3.68 (95% CI, 2.82–4.79, *p*  < 0.001, Table 5), 1.69 (95% CI, 1.03–2.76, *p*=0.037, Table 5), and 2.69 (95% CI, 1.58–4.56, *p*  < 0.001, Table 5), respectively. Moreover, in alignment with the results from the training set, patients classified in the high-risk group exhibited an elevated risk of AKI, new AKI, persistent AKI, and AKI progression in both the internal and external validation sets, despite adjustments for multiple variables (Supporting Information Table S3). The results demonstrated that our innovative WBC-related indicators risk model shown effective performance in predicting AKI.

### 3.4. The Novel Model's Performance in ICU Mortality and In-Hospital Mortality Prediction

**Figure 5:**
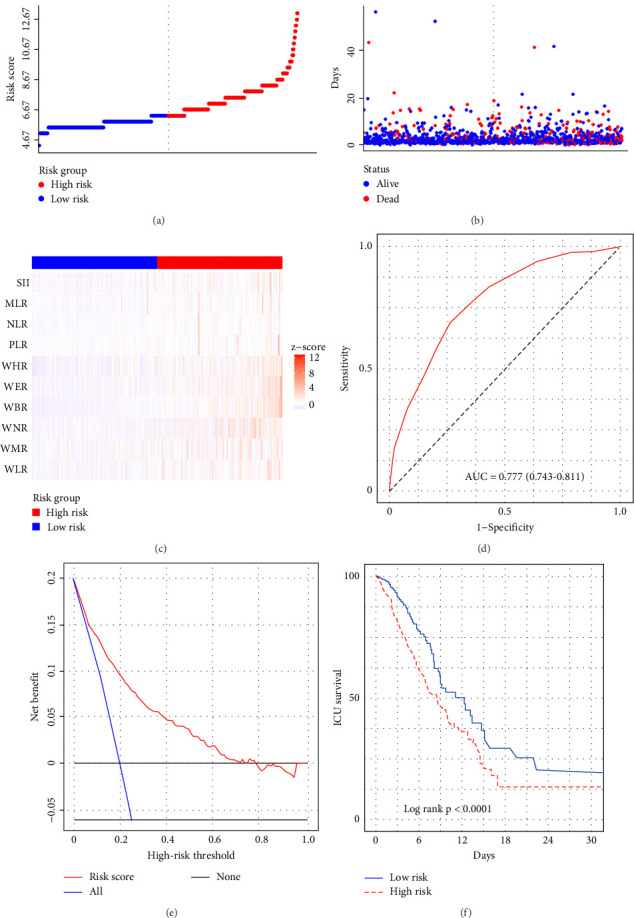
The risk score was established to detect the intensive care unit mortality of critically ill hepatocellular carcinoma patients in the training set. All patients were distinguished into high and low risk based on the risk score, (A, B) the relationship between survival time and prognosis of patients in the two corresponding groups, and the (C) heatmap of inflammatory marks between the two groups. (D) Receiver operating characteristic (ROC) curve analysis of the risk score for in-hospital mortality. (E) Decision curve analysis of the risk score for in-hospital mortality. (F) Kaplan–Meier curves showing the in-hospital mortality of groups with different risk.

**Figure 6:**
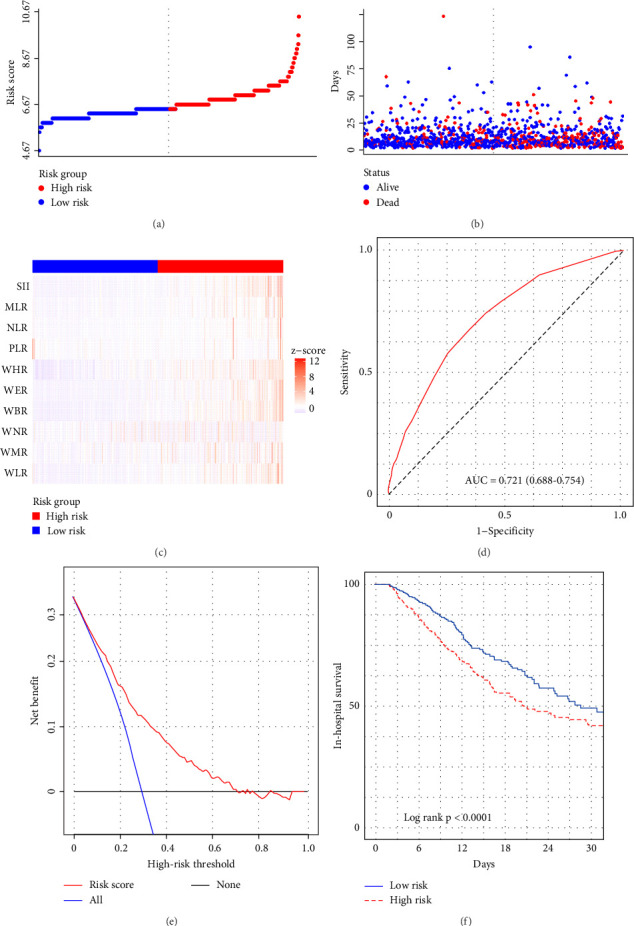
The risk score was established to detect the in-hospital mortality of critically ill hepatocellular carcinoma patients in the training set. All patients were distinguished into high and low risk based on the risk score, (A, B) the relationship between survival time and prognosis of patients in the two corresponding groups and the (C) heatmap of inflammatory marks between the two groups. (D) Receiver operating characteristic (ROC) curve analysis of the risk score for in-hospital mortality. (E) Decision curve analysis of the risk score for in-hospital mortality. (F) Kaplan–Meier curves showing the in-hospital mortality of groups with different risk.

We subsequently evaluated the efficacy of the unique WBC-related indicators-based risk model in predicting ICU mortality and in-hospital mortality in critically ill HCC patients, excluding AKI prediction. A crude OR of 1.88 (95% CI, 1.41–2.51, *p*  < 0.001) for ICU mortality and 1.52 (95% CI, 1.21–1.90, *p*  < 0.001) for in-hospital mortality was seen in the high-risk group, with the connection persisting robustly after adjusting for multiple risk factors in the training set (Table 6). The findings from both the internal and external validation cohorts corroborated the claims of the training set (Supporting Information Table S4). Additionally, ROC analysis was conducted to evaluate the predictive efficacy of the risk score in comparison to the SOFA score, OASIS score, APSIII score, SAPSII score, and Charlson index regarding ICU mortality and in-hospital mortality. The findings demonstrated that the innovative risk score displayed superior predictive capability for in-hospital mortality relative to the SOFA score, OASIS score, APSIII score, SAPSII score, and Charlson index (Supporting Information Figure S2). Figures 5A–C and 6A–C demonstrate that the likelihood of ICU death and in-hospital mortality escalates with higher risk scores. Figures 5D and 6D indicate that the area under the curve (AUC) for the risk score predicting ICU mortality was 0.777 (95% CI, 0.743–0.811) and 0.721 (95% CI, 0.688–0.754), respectively. The risk model demonstrated clinical utility in forecasting ICU and in-hospital mortality, as indicated by DCA (Figures 5E, 6E). Furthermore, in nearly all subgroups within both the internal and external validation sets, the pre-defined subgroup analysis indicated that high-risk patients exhibited a greater likelihood of ICU death and in-hospital mortality compared to their low-risk counterparts (Supporting Information Figure S3). Furthermore, Kaplan–Meier curve analysis indicated that the prognosis for the high-risk group was inferior to that of the low-risk group (Figures 5F and 6F). Patients from both the internal and external validation sets exhibited comparable outcomes (Supporting Information Figure S4 and S5), thereby corroborating the predictive efficacy of the novel risk model for ICU and in-hospital mortality.

## 4. Discussion

The AKI-prediction model utilizing leukocyte-related factors has not been thoroughly investigated in critically sick HCC contexts. This study focused on leukocyte-related factors and created an innovative risk model including indicators such as WNR, WMR, WHR, and PLR. We evaluated the predictive accuracy of a novel risk model for AKI in patients with HCC admitted to the ICU, comparing it to established risk scoring models (SOFA, OASIS, APS III, SAPS II, and Charlson index). The results indicated that the novel risk model effectively predicted AKI. Moreover, individuals classified in the high-risk group had an elevated likelihood of AKI, new-onset AKI, persistent AKI, and progression of AKI compared to those in the low-risk group. We additionally evaluated the risk model's ability to predict both ICU and in-hospital mortality. The results indicated that our risk model demonstrated a robust history in forecasting AKI, as well as ICU and inpatient mortality. Our leukocyte-related indicators model was employed to develop a risk estimator that assesses the probability of AKI in high-risk patients with critically ill HCC.

A common side effect of HCC patients is AKI. After liver transplantation, 19.26% of HCC patients had AKI, according to Chen's research [20]. 11.0% of HCC patients having transarterial chemoembolization, according to Sohn et al., [21] suffered AKI. Following hepatectomy for HCC, 15% of patients experienced AKI, according to Lim et al. [8]. In this study, AKI occurred in 23.2% of patients at our institution, 35.8% of patients from the MIMIC-IV database, and 17.9% of patients from the eICU-CRD database. Due to the presence of HCC patients in ICU settings, the incidence of AKI was higher than previously documented. It is unclear what potential mechanisms may be at play when critically ill HCC patients cause AKI. AKI brought on by HCC may be explained by hepatorenal syndrome (HRS). Patients' reversible AKI is described by HRS. The sympathetic nervous system (SNS) and the renin–angiotensin-aldosterone system (RAAS) are overactive as a result of severe HCC, which also causes vasoconstriction, structural damage to the kidney, intravascular hypovolemia, necrosis, and apoptosis of tubular cells, which ultimately results in a complete decline in the GFR [22, 23].

Additionally, it was discovered that in patients with HCC, AKI, and AKI progression, the inflammatory response was a significant predictor of disease progression and prognosis [24, 25]. Systemic immune-inflammation index (SII), an inflammatory biomarker, was found to be a reliable predictor of postoperative AKI in HCC patients in a prior study [11]. However, prior research with relation to critically ill HCC-associated AKI had a limited understanding of the prognostic capacity of WBCs as an overall notion of inflammatory markers. The goal of the current investigation was to identify possible WBC-related indicators and then incorporate these factors into a risk model to forecast AKI associated with critically sick HCC. A WBC-related indicators-based risk model for prognosis prediction has been published in earlier investigations. Preoperative WHR, WMR, and PLR have already been linked to bladder cancer prognosis by Gao et al. [26] A risk model based on WHR, WMR, and PLR has also demonstrated high prognosis predictive ability. Another study looked into using WBC-related indicators to predict AKI in critically ill ischemic stroke patients. The findings showed that the risk model built using WLR, WBR, WHR, and neutrophil to lymphocyte ratio (NLR) performed well at predicting AKI in these patients. The current study discovered that WNR, WMR, WHR, and PLR were independent predictors for AKI prediction, and WNR, WMR, WHR, and PLR were merged to create a novel risk model. This finding is consistent with other reports [27]. In addition, except for predicting AKI, the novel WBC-related indicators risk model also exhibited good mortality predictive performance.

As is well known, systemic inflammation plays a crucial role in the occurrence and metastasis of cancer. Research over the past decade has confirmed that systemic inflammatory markers can predict the prognosis and postoperative recurrence of various tumors, including HCC. Various inflammatory cells, such as neutrophils, platelets, and monocytes, have also been shown to be associated with the prognosis of HCC [28, 29]. Several studies have verified that NLR, PLR, and lymphocyte to monocyte ratio (LMR) are predictive factors for HCC prognosis prediction [30]. Moreover, inflammation is associated with AKI. And leukocyte-related biomarkers were reported to predict AKI in different disease conditions [31, 32]. Hence, the potential mechanism by which inflammatory response may lead to AKI in HCC, and the use of leukocyte-related indicators risk model to predict AKI in HCC is reasonable, but further research is needed to confirm this result in the future.

This study presented multiple limitations. The retrospective nature of the study led to insufficient clinical data, which hindered the inclusion of numerous patients and introduced potential selection bias. Second, we could not obtain specific essential information concerning the type and treatment of the HCC. Third, the use of diuretics can lead to inaccuracies in urine volume data; therefore, the urine standard was not utilized in the diagnosis of AKI. The overall incidence rate of AKI may decrease as a consequence. We evaluated the changes in WBC-related variables solely after ICU admission, excluding the hospital stay period. Static measurement of WBC-related variables may fail to accurately reflect the inflammatory and immunological status of patients without dynamic monitoring. Given the absence of high-quality prognosis prediction tools for critically ill HCC patients, our innovative risk model may aid clinicians in developing accurate risk assessments and treatment strategies. While the models developed through logistic regression and Cox regression are adequate, it is advisable to explore alternative modeling techniques, such as support vector machines (SVM) or XGBoost, to enhance predictive performance.

## 5. Conclusions

A novel prognostic predictive risk model was developed in this study, utilizing WBC-related indicators, including WNR, WMR, WHR, and PLR. The risk model demonstrated effective performance in predicting acute kidney injury and mortality. We assert that additional research, particularly large prospective studies, should corroborate our findings.

## Figures and Tables

**Table 1:** Comparisons of characteristics between no AKI group and AKI group.

Characteristics	Training set (*n* = 1060)			Internal validation set (*n* = 469)		
	No AKI (*n* = 681)	AKI (*n* = 379)	*p* Value	No AKI (*n* = 385)	AKI (*n* = 84)	*p* Value
Age, years old	65.9 ± 12.2	65.7 ± 11.7	0.833	63.9 ± 12.0	63.6 ± 12.8	0.803
Gender, male (*n*, %)	378 (55.5)	269 (71.0)	**<0.001**	177 (46.0)	42 (50.0)	0.583
Weight (kg)	74.6 ± 18.4	82.7 ± 21.4	**<0.001**	80.4 ± 22.0	82.2 ± 24.4	0.495
Ethnicity (*n*, %)	—	—	0.264	—	—	0.608
White	489 (71.8)	254 (67.0)	—	281 (73.0)	60 (71.4)	—
Black	81 (11.9)	53 (14.0)	—	79 (20.5)	16 (19.0)	—
Others	111 (16.3)	72 (19.0)	—	25 (6.5)	8 (9.5)	—
Comorbidities (*n*, %)	—	—	—	—	—	—
Myocardial infarction	61 (9.0)	28 (7.4)	0.443	15 (3.9)	8 (9.5)	0.059
Congestive heart failure	101 (14.8)	63 (16.6)	0.494	27 (7.0)	8 (9.5)	0.573
Hypertension	246 (36.1)	135 (35.6)	0.923	172 (44.7)	37 (44.0)	1.000
Diabetes	163 (23.9)	110 (29.00	0.081	104 (27.0)	4 (4.8)	0.610
Chronic kidney disease	87 (12.8)	63 (16.6)	0.103	27 (7.0)	4 (4.8)	0.610
Other liver disease	196 (28.8)	188 (49.6)	**<0.001**	39 (10.1)	8 (9.5)	1.000
CCI, points	10.0 ± 2.6	10.0 ± 2.6	0.875	7.8 ± 3.8	7.7 ± 3.2	0.771
Drugs usage (*n*, %)	—	—	—	—	—	—
ACEI/ARB	96 (14.1)	54 (14.2)	1.000	64 (16.6)	14 (16.7)	1.000
* β* blockers	288 (42.3)	185 (48.8)	**0.047**	145 (37.7)	31 (36.9)	0.996
Calcium channel blockers	71 (10.4)	63 (16.6)	**0.005**	35 (9.1)	12 (32.1)	0.701
Score system, points	—	—	—	—	—	—
SOFA	4.9 ± 2.3	9.1 ± 4.2	**<0.001**	4.6 ± 1.5	5.8 ± 2.3	**<0.001**
OASIS	31.1 ± 7.8	36.7 ± 9.3	**<0.001**	21.7 ± 9.6	25.0 ± 10.4	**0.009**
APSIII	48.6 ± 21.3	73.7 ± 27.6	**<0.001**	45.0 ± 21.8	54.7 ± 27.1	**<0.001**
SAPSII	41.2 ± 11.9	53.9 ± 15.5	**<0.001**	—	—	—
Vital signs	—	—	—	—	—	—
MAP (mmHg)	82.8 ± 16.4	76.7 ± 18.6	**<0.001**	85.1 ± 20.0	88.7 ± 16.5	0.123
Heart rate (bpm)	97.4 ± 20.3	97.7 ± 22.4	0.856	84.4 ± 20.6	96.1 ± 21.1	0.487
RR (bpm)	20.0 ± 5.6	21.0 ± 6.8	**0.006**	20.1 ± 6.2	19.7 ± 6.3	0.660
SpO_2_ (%)	96.5 ± 4.1	96.2 ± 3.9	0.253	96.8 ± 3.0	96.7 ± 3.2	0.716
Laboratory values	—	—	—	—	—	—
White blood cell, ×10^9^/L	11.6 ± 4.9	11.5 ± 4.8	0.764	11.5 ± 4.3	12.7 ± 5.3	0.206
Hemoglobin (g/dL)	10.1 ± 2.1	10.4 ± 2.2	0.023	10.6 ± 2.3	11.0 ± 2.7	0.135
Platelet, ×10^9^/L	206.6 ± 101.1	196.1 ± 98.9	0.236	213.3 ± 103.5	205.9 ± 100.6	0.642
Albumin (g/dL)	3.1 ± 0.6	2.8 ± 0.6	**<0.001**	2.9 ± 0.7	2.4 ± 0.8	<0.001
Bilirubin (mmol/L)	2.3 ± 1.3	4.1 ± 1.6	**<0.001**	2.5 ± 1.4	3.6 ± 1.7	0.038
Anion gap (mEq/L)	14.8 ± 3.9	15.4 ± 4.8	**0.021**	11.5 ± 5.1	12.3 ± 5.1	0.225
Bicarbonate (mEq/L)	23.0 ± 4.6	22.2 ± 4.7	0.013	23.2 ± 5.2	23.2 ± 5.2	0.962
BUN (mg/dL)	24.6 ± 9.2	30.7 ± 11.8	**<0.001**	25.6 ± 11.9	36.0 ± 10.9	**<0.001**
Creatinine (mg/dL)	1.1 ± 0.5	1.5 ± 0.6	**<0.001**	1.2 ± 0.4	1.6 ± 0.5	**<0.001**
Potassium (mmol/L)	4.2 ± 0.7	4.4 ± 0.9	**<0.001**	4.3 ± 0.8	4.3 ± 0.9	0.703
Sodium (mmol/L)	136.3 ± 5.6	135.9 ± 5.2	0.239	135.0 ± 6.4	135.8 ± 5.5	0.309

*Note*: Bold indicates a *p*-value less than 0.05.

Abbreviations: AKI, acute kidney injury; APSIII, acute physiology score III; BUN, blood urea nitrogen; CCI, Charlson comorbidity index; MAP, mean arterial pressure; OASIS, oxford acute severity of illness score; RR, respiratory rate; SAPSII, simplified Acute Physiology Score II; SIRS, systemic inflammatory response syndrome; SOFA, sequential organ failure assessment; SpO_2_, saturation of peripheral oxygen.

**Table 2:** Logistic regression analysis of WBC-related indicators for AKI in training set.

	Univariate			Multivariate		
	*β*	OR (95% CI)	*p*	*β*	OR (95% CI)	*p*
WNR	1.154	3.17 (2.52–3.99)	<0.001	1.220	3.39 (2.66–4.32)	**<0.001**
WMR	0.021	1.02 (1.01–1.03)	<0.001	—	—	—
Lg (WMR)	1.303	3.68 (2.30–5.90)	<0.001	1.031	2.81 (1.48–5.32)	**0.002**
WLR	0.019	1.02 (1.01–1.03)	<0.001	—	—	—
Lg (WLR)	0.985	2.68 (1.83–3.91)	<0.001	0.020	1.02 (0.51–2.04)	0.955
WER	0.004	1.00 (1.00–1.01)	<0.001	—	—	—
Lg (WER)	0.275	1.32 (1.11–1.56)	<0.001	−0.186	0.83 (0.65–1.46)	0.653
WBR	0.005	1.01 (1.00–1.01)	<0.001	—	—	—
Lg (WBR)	0.759	2.14 (1.62–2.81)	<0.001	0.274	1.32 (0.86–2.00)	0.201
WHR	0.808	2.24 (1.84–2.74)	<0.001	0.970	2.64 (1.97–3.54)	**<0.001**
NLR	0.026	1.03 (1.02–1.04)	<0.001	0.011	1.01 (0.98–1.04)	0.176
PLR	0.001	1.00 (1.00–1.00)	<0.001	—	—	—
Lg (PLR)	0.593	1.81 (1.34–2.45)	<0.001	0.870	2.39 (1.56–3.64)	**<0.001**
MLR	0.061	1.06 (0.99–1.13)	0.052	—	—	—
SII	0.011	1.01 (1.00–1.02)	0.013	—	—	—
Lg (SII)	0.321	1.38 (1.12–1.70)	0.002	−0.128	0.88 (0.60–1.30)	0.522

*Note*: Bold indicates a *p*-value less than 0.05.

Abbreviations: AKI, acute kidney injury; OR, odds ratio; NLR, neutrophil to lymphocyte ratio; 95%CI, 95% confidence index; MLR, monocyte to lymphocyte ratio; PLR, platelets to lymphocyte ratio; SII, systemic immune-inflammation index; WBC, white blood count; WBR, WBC to basophils ratio; WER, WBC to eosinophils ratio; WHR, WBC to hemoglobin ratio; WLR, WBC to lymphocyte ratio; WMR, WBC to monocyte ratio; WNR, WBC to neutrophil ratio.

**Table 3:** Change in serum creatinine before and after the diagnosis of AKI for AKI patients.

Variables	High-risk group	Low-risk group	*p* Value
In training set	—	—	—
Serum creatinine at ICU admission, mean ± SD	1.58 ± 0.71	1.41 ± 0.65	**0.046**
Serum creatinine at ICU admission, median (IQR)	1.30 (1.90, 1.88)	1.10 (0.80, 1.60)	—
Serum creatinine at first AKI diagnosis, mean ± SD	2.36 ± 1.56	2.00 ± 0.92	**0.041**
Serum creatinine at first AKI diagnosis, median (IQR)	1.80 (1.30, 3.10)	1.60 (1.10, 2.40)	—
Serum creatinine at 48 h after AKI diagnosis, mean ± SD	2.65 ± 1.30	2.10 ± 1.15	**<0.001**
Serum creatinine at 48 h after AKI diagnosis, median (IQR)	1.90 (1.40, 3.30)	1.60 (1.20, 2.60)	—
Positive changes of serum creatinine (*n*) (%)	150 (51.4)	43 (49.4)	—
ΔSerum creatinine, mean ± SD	0.87 ± 0.69	0.41 ± 0.28	**<0.001**
Negative or static change of serum creatinine (*n*) (%)	142 (48.6)	44 (50.6)	—
ΔSerum creatinine, mean ± SD	−0.41 ± 0.30	−0.43 ± 0.26	0.689
In internal validation set	—	—	—
Serum creatinine at ICU admission, mean ± SD	1.83 ± 0.96	1.46 ± 0.69	**0.046**
Serum creatinine at ICU admission, median (IQR)	1.35 (0.99, 2.17)	1.24 (0.83, 1.94)	—
Serum creatinine at first AKI diagnosis, mean ± SD	2.52 ± 1.19	1.85 ± 0.94	**0.015**
Serum creatinine at first AKI diagnosis, median (IQR)	2.28 (1.51, 2.92)	1.79 (1.09, 2.64)	—
Serum creatinine at 48 h after AKI diagnosis, mean ± SD	2.10 ± 0.98	1.51 ± 0.73	**0.002**
Serum creatinine at 48 h after AKI diagnosis, median (IQR)	1.79 (1.21, 2.92)	1.44 (0.81, 2.08)	—
Positive changes of serum creatinine (*n*) (%)	21 (50.0)	17 (40.5)	—
ΔSerum creatinine, mean ± SD	0.90 ± 1.12	0.55 ± 0.61	0.256
Negative or static change of serum creatinine (*n*) (%)	21 (50.0)	25 (59.5)	—
ΔSerum creatinine, mean ± SD	−0.54 ± 0.46	−0.44 ± 0.58	0.526

*Note*: Bold indicates a *p*-value less than 0.05.

Abbreviations: AKI, acute kidney injury; ICU, intensive care unit; IQR, interquartile range; SD, standard deviation.

**Table 4:** Clinical outcomes analysis of high- and low-risk groups for patients in training set.

Outcomes	Low-risk group	High-risk group	Effect size	*p* Value
*N*	575	485	—	—
Primary outcome	—	—	—	—
AKI	87 (15.1)	292 (60.2)	1.051	**<0.001**
Secondary outcomes	—	—	—	—
AKI severity^a^	—	—	1.053	**<0.001**
Stage I	53 (60.9)	162 (55.5)	—	—
Stage II	21 (24.1)	71 (24.3)	—	—
Stage III	13 (14.9)	59 (20.2)	—	—
Days between ICU admission and AKI^a^	0.98 ± 0.47	1.02 ± 0.45	0.688	**<0.001**
New AKI^b^	48 (9.8)	30 (15.5)	1.062	**<0.001**
Persistent AKI^a^	55 (63.2)	240 (82.2)	1.076	**<0.001**
AKI progression^a^	26 (29.9)	128 (43.8)	1.061	**<0.001**
Usage of CRRT	11 (1.9)	32 (6.6)	0.234	**<0.001**
Usage of vasopressor	162 (28.2)	213 (43.9)	0.332	**<0.001**
Usage of mechanical ventilation	190 (33.0)	195 (40.2)	0.149	**0.019**
Usage of diuretic	209 (53.7)	278 (57.3)	0.269	0.072
Acute heart failure	41 (7.1)	31 (6.4)	0.029	0.724
Acute respiratory failure	75 (13.0)	90 (18.6)	0.152	**0.017**
Acute hepatic failure	31 (5.4)	45 (9.3)	0.150	**0.020**
Sepsis	307 (53.4)	324 (66.8)	0.277	**<0.001**
Length of ICU stay	1.92 (1.08, 3.72)	2.55 (1.51, 4.44)	0.113	**0.068**
Length of hospital stay	8.40 (5.60, 14.60)	10.00 (6.10, 16.80)	0.134	**0.029**
In-hospital mortality	128 (22.3)	189 (39.0)	0.403	**<0.001**
ICU mortality	72 (12.5)	138 (28.5)	0.369	**<0.001**

*Note*: Bold indicates a *p*-value less than 0.05.

Abbreviations: AKI, acute kidney injury; CRRT, continues renal replacement therapy; ICU, intensive care unit.

^a^Excluded patients without the incidence of AKI.

^b^Excluded patients with the incidence of AKI.

**Table 5:** Univariate and multivariate logistic regression analysis for clinical outcomes in training set.

Methods	OR (95%CI)	*p* Value
For AKI	—	—
Unadjusted	3.68 (2.82–4.79)	**<0.001**
Adjusted for model I	3.97 (2.01–5.24)	**<0.001**
Adjusted for model II	4.03 (2.89–5.63)	**<0.001**
Adjusted for model III	3.60 (2.52–5.14)	**<0.001**
For new AKI^a^	—	—
Unadjusted	1.69 (1.03–2.76)	**0.037**
Adjusted for model I	1.78 (1.08–2.94)	**0.024**
Adjusted for model II	1.88 (1.06–3.33)	**0.032**
Adjusted for model III	2.15 (1.05–4.42)	**0.037**
For persistent AKI^b^	—	—
Unadjusted	2.69 (1.58–4.56)	**<0.001**
Adjusted for model I	2.79 (1.63–4.78)	**<0.001**
Adjusted for model II	3.13 (1.82–6.04)	**<0.001**
Adjusted for model III	3.74 (1.87–7.49)	**<0.001**
For AKI progression^b^	—	—
Unadjusted	1.83 (1.10–3.06)	**0.021**
Adjusted for model I	2.21 (1.23–4.97)	**0.008**
Adjusted for model II	2.20 (1.16–4.14)	**0.015**
Adjusted for model III	2.15 (1.05–4.42)	**0.037**

*Note:* Model I adjusted for age, gender, weight, and ethnicity. Model II adjusted for model I plus comorbidities and Charlson comorbidity index, score system, interventions, and drug usage. Model III adjusted for model II plus vital signs and laboratory results except for white blood count. Bold indicates a *p*-value less than 0.05.

Abbreviations: AKI, acute kidney injury; OR, odds ratio; 95%CI, 95% confidence index.

^a^Excluded patients with the incidence of AKI.

^b^Excluded patients without the incidence of AKI.

**Table 6:** Univariate and multivariate Cox regression analysis for clinical outcomes in training set.

Methods	HR (95% CI)	*p* Value
For ICU mortality	—	—
Unadjusted	1.88 (1.41–2.51)	**<0.001**
Adjusted for model I	1.91 (1.43–2.55)	**<0.001**
Adjusted for model II	1.86 (1.24–2.32)	**0.012**
Adjusted for model III	1.75 (1.29–2.39)	**0.034**
For in-hospital mortality	—	—
Unadjusted	1.52 (1.21–1.90)	**<0.001**
Adjusted for model I	1.87 (1.51–2.89)	**<0.001**
Adjusted for model II	2.19 (1.33–2.52)	**0.011**
Adjusted for model III	2.02 (1.18–4.33)	**0.037**

*Note:* Model I adjusted for age, gender, weight, and ethnicity. Model II adjusted for model I plus comorbidities and Charlson comorbidity index, HAS-BLED score, score system, interventions, and drug usage. Model III adjusted for model II plus vital signs and laboratory results except for white blood count. Bold indicates a *p*-value less than 0.05.

Abbreviations: AKI, acute kidney injury; HR, hazard ratio; 95%CI, 95% confidence index.

## Data Availability

The datasets used are available from the corresponding author on reasonable request.

## Supporting Information

Supporting Information Supporting Information Table S1: Comparisons of characteristics between no AKI group and AKI group in external validation set. Supporting Information Table S2: Clinical outcomes analysis of high- and low-risk groups for patients in validation sets. Supporting Information Table S3: Univariate and multivariate logistic regression analysis for clinical outcomes in validation sets. Supporting Information Table S4: Univariate and multivariate Cox regression analysis for clinical outcomes in validation sets. Supporting Information Figure S1: The waterfall plots of the high-risk group and low-risk group for the prediction of AKI (A), new AKI (B), AKI progression (C), and persistent AKI (D) for patients in the internal validation set. Supporting Information Figure S2. ROC curves of risk score and severity score for the prediction of (A) ICU mortality and (B) in-hospital mortality among critically ill HCC patients. Supporting Information Figure S3. The waterfall plots and forest plots of the risk score in individuals with HCC for ICU mortality and in-hospital mortality in the training set. Supporting Information Figure S4. The risk score was established to detect the ICU and in-hospital mortality of patients with HCC in the internal validation set. All patients were distinguished into high and low risk based on the risk score (A), the relationship between survival time and prognosis of patients in the two corresponding groups (B), and the heatmap of inflammatory marks between the two groups (C). The Kaplan–Meier curves showing the in-hospital mortality (D) and ICU mortality (E) of groups with different risk. The risk score was established to detect the ICU mortality of patients with liver cancer in the internal validation set. All patients were distinguished into high and low risk based on the risk score (F), the relationship between survival time and prognosis of patients in the two corresponding groups (G), and the heatmap of inflammatory marks between the two groups (H). Supporting Information Figure S5. The waterfall plots and the Kaplan–Meier curves of the risk score in individuals with HCC for clinical outcomes in the external validation set. The waterfall plots of the high-risk group and low-risk group for the prediction of AKI (A), ICU mortality (B), and in-hospital (C) for patients in the external validation set. The Kaplan–Meier curves showing the ICU mortality (D) and in-hospital mortality (E) of groups with different risk.
