# Supplementary material for: A Predictive Model for Acute Kidney Injury Based on Leukocyte-Related Indicators in Hepatocellular Carcinoma Patients Admitted to the Intensive Care Unit
Source: Mediators Inflamm. 2025 Apr 16;2025:7110012. doi: 10.1155/mi/7110012 (PMC12017962; doi:10.1155/mi/7110012)
Supplement: Supporting Information — Table S1: Comparisons of characteristics between no AKI group and AKI group in external validation set. Supporting Information Table S2: Clinical outcomes analysis of high- and low-risk groups for patients in validation sets. Supporting Information Table S3: Univariate and multivariate logistic regression analysis for clinical outcomes in validation sets. Supporting Information Table S4: Univariate and multivariate Cox regression analysis for clinical outcomes in validation sets. Supporting Information Figure S1: The waterfall plots of the high-risk group and low-risk group for the prediction of AKI (A), new AKI (B), AKI progression (C), and persistent AKI (D) for patients in the internal validation set. Supporting Information Figure S2. ROC curves of risk score and severity score for the prediction of (A) ICU mortality and (B) in-hospital mortality among critically ill HCC patients. Supporting Information Figure S3. The waterfall plots and forest plots of the risk score in individuals with HCC for ICU mortality and in-hospital mortality in the training set. Supporting Information Figure S4. The risk score was established to detect the ICU and in-hospital mortality of patients with HCC in the internal validation set. All patients were distinguished into high and low risk based on the risk score (A), the relationship between survival time and prognosis of patients in the two corresponding groups (B), and the heatmap of inflammatory marks between the two groups (C). The Kaplan–Meier curves showing the in-hospital mortality (D) and ICU mortality (E) of groups with different risk. The risk score was established to detect the ICU mortality of patients with liver cancer in the internal validation set. All patients were distinguished into high and low risk based on the risk score (F), the relationship between survival time and prognosis of patients in the two corresponding groups (G), and the heatmap of inflammatory marks between the two groups (H). [file 7110012.f1.docx]

**Supplemental Table 1 Comparisons of characteristics between no AKI group and AKI group in external validation set**

|  | No AKI (n=76) | AKI (n=23) | P value |
| --- | --- | --- | --- |
| Age, years old | 60.4 ± 12.3 | 64.3± 10.8 | 0.171 |
| Gender, male, n (%) | 60 (78.9) | 18 (78.3) | 1.000 |
| Weight, kg | 67.8 ± 11.4 | 66.1 ± 12.7 | 0.552 |
| Ethnicity, n (%) |  |  | 1.000 |
| White | 0 (0.0) | 0 (0.0) |  |
| Black | 0 (0.0) | 0 (0.0) |  |
| Others | 76 (100.0) | 23 (100.0) |  |
| Comorbidities, n (%) |  |  |  |
| Myocardial infarction | 3 (3.9) | 1 (4.3) | 1.000 |
| Congestive heart failure | 2 (2.6) | 4 (17.4) | 0.036 |
| Hypertension | 9 (11.8) | 3 (13.0) | 1.000 |
| Diabetes | 11 (14.5) | 2 (8.7) | 0.714 |
| Chronic kidney disease | 5 (6.6) | 5 (21.7) | 0.086 |
| Other liver disease | 24 (31.6) | 8 (34.8) | 0.973 |
| CCI, points | 6.0 ± 2.1 | 5.7 ± 1.8 | 0.607 |
| Drugs usage, n (%) |  |  |  |
| ACEI/ARB | 5 (6.6) | 2 (8.7) | 1.000 |
| β blockers | 3 (3.9) | 3 (13.0) | 0.270 |
| Calcium channel blockers | 2 (2.6) | 0 (0.0) | 1.000 |
| Score system, points |  |  |  |
| SOFA | 3.1 ± 1.4 | 4.8 ± 1.5 |  |
| OASIS | 20.9 ± 5.4 | 31.5 ± 8.6 |  |
| APSIII | 33.7 ± 11.5 | 48.2 ± 14.8 |  |
| SAPSII | 32.1 ± 16.9 | 48.4 ± 16.6 |  |
| Vital signs |  |  |  |
| MAP, mmHg | 90.8 ± 28.6 | 79.8 ± 17.6 | 0.083 |
| Heart rate, bpm | 92.0 ± 19.9 | 91.4 ± 13.8 | 0.893 |
| RR, bpm | 18.5 ± 4.5 | 19.7 ± 6.1 | 0.295 |
| SpO_2_, % | 98.3 ±2.4 | 95.7 ± 4.9 | ≤0.001 |
| Laboratory values |  |  |  |
| White blood cell, × 10^9^/L | 9.4 ± 2.8 | 10.9 ± 3.2 | 0.392 |
| Hemoglobin, g/dL | 9.3 ± 3.1 | 8.2 ± 1.8 | 0.138 |
| Platelet, × 10^9^/L | 115.2 ± 49.2 | 122.9 ± 58.0 | 0.696 |
| Albumin, g/dL | 3.0 ± 0.7 | 2.5 ± 0.8 | 0.017 |
| Bilirubin, mmol/L | 2.7 ± 1.3 | 4.2 ± 2.1 | 0.291 |
| Anion gap, mEq/L | 14.1 ± 3.7 | 16.1 ± 4.7 | 0.039 |
| Bicarbonate, mEq/L | 21.1 ± 6.3 | 18.3 ± 7.4 | 0.078 |
| BUN, mg/dL | 10.3 ± 3.1 | 18.4 ± 6.9 | <0.001 |
| Creatinine, mg/dL | 0.8 ± 0.3 | 2.0 ± 1.0 | <0.001 |
| Potassium, mmol/L | 4.4 ± 1.4 | 4.0 ± 0.9 | 0.773 |
| Sodium, mmol/L | 135.5 ± 6.9 | 137.9 ± 3.9 | 0.403 |

AKI, acute kidney injury, CCI, Charlson comorbidity index, SOFA, sequential organ failure assessment, OASIS, oxford acute severity of illness score, APSIII, acute physiology score III, SAPSII, simplified Acute Physiology Score II, SIRS, systemic inflammatory response syndrome, MAP, mean arterial pressure, RR, respiratory rate, SpO2, saturation of peripheral oxygen, BUN, blood urea nitrogen.

**Supplemental Table 2 Clinical outcomes analysis of high and low risk groups for patients in validation sets**

| Outcomes | Internal validation set | | | | External validation set | | | |
| --- | --- | --- | --- | --- | --- | --- | --- | --- |
|  | Low risk | High risk | Effect size | P | Low risk | High risk | Effect size | P |
| N | 359 | 110 | - | - | 74 | 25 |  |  |
| Primary outcome |  |  |  |  |  |  |  |  |
| AKI | 42 (11.7) | 42 (38.2) | 0.643 | **<0.001** | 10 (13.5) | 13 (52.0) | 1.199 | **<0.001** |
| Secondary outcomes |  |  |  |  |  |  |  |  |
| AKI severity^1^ |  |  | 0.681 | **<0.001** |  |  | 1.243 | **<0.001** |
| Stage I | 36 (85.7) | 25 (59.5) |  |  | 7 (70.0) | 4 (30.8) |  |  |
| Stage II | 2 (4.8) | 9 (21.4) |  |  | 2 (20.0) | 4 (30.8) |  |  |
| Stage III | 4 (9.5) | 8 (19.1) |  |  | 1 (10.0) | 5 (38.4) |  |  |
| Days between ICU admission and AKI^1^ | 0.52 (0.33) | 0.67 (0.42) | 0.262 | **0.013** | 0.50 (0.36) | 0.73 (0.60) | 0.300 | 0.504 |
| New AKI^2^ | 45 (14.2) | 23 (33.8) | 0.783 | **<0.001** | 7 (10.6) | 4 (40.0) | 0.718 | **0.048** |
| Persistent AKI^1^ | 21 (50.0) | 31 (73.8) | 0.674 | **<0.001** | 3 (37.5) | 11 (73.3) | 0.773 | 0.219 |
| AKI progression^1^ | 12 (28.6) | 21 (50.0) | 0.665 | **<0.001** | 1 (12.5) | 10 (66.7) | 1.330 | **0.041** |
| CRRT | 8 (2.2) | 3 (2.7) | 0.032 | 1.000 | 14 (18.9) | 12 (48.0) | 0.648 | **0.009** |
| Vasopressors | 61 (17.0) | 23 (20.9) | 0.100 | 0.426 | 39 (52.7) | 17 (68.0) | 0.317 | 0.271 |
| MV | 87 (24.2) | 34 (30.9) | 0.150 | 0.202 | 29 (39.2) | 17 (68.0) | 0.603 | **0.023** |
| Diuretic | 122 (34.0) | 40 (36.4) | 0.050 | 0.730 | 15 (20.3) | 7 (28.0) | 0.181 | 0.599 |
| AHF | 12 (3.3) | 6 (5.5) | 0.103 | 0.468 | 10 (13.5) | 8 (32.0) | 0.452 | 0.076 |
| ARF | 83 (23.1) | 35 (31.8) | 0.196 | 0.087 | 5 (6.8) | 6 (24.0) | 0.492 | **0.045** |
| Sepsis | 167 (46.5) | 71 (64.5) | 0.369 | **≤0.001** | 29 (39.2) | 17 (68.0) | 0.603 | **0.023** |
| LOS of ICU | 2.1 (1.2, 3.9) | 2.1 (0.9, 3.9) | 0.034 | 0.744 | 2.0 (1.0,3.0) | 2.0 (1.0, 4.0) | 0.454 | 0.184 |
| LOS of hospital | 6.9 (4.7, 10.9) | 7.9 (4.8, 13.2) | 0.166 | **0.052** | 7.0 (3.5, 12.0) | 6.0 (5.0, 12.0) | 0.328 | 0.248 |
| In-hospital death | 53 (14.8) | 40 (36.4) | 0.511 | **<0.001** | 8 (10.8) | 9 (36.0) | 0.623 | **0.010** |
| ICU death | 20 (5.6) | 13 (11.8) | 0.233 | **0.043** | 5 (6.8) | 8 (32.0) | 0.674 | **0.004** |

AKI, acute kidney injury, ICU, intensive care unit, CRRT, continues renal replacement therapy.

^1^Excluded patients without the incidence of AKI.

^2^Excluded patients with the incidence of AKI.

**Supplemental Table 3 Univariate and multivariate logistic regression analysis for clinical outcomes in validation sets**

| Methods | Internal validation set | | External validation set | |
| --- | --- | --- | --- | --- |
|  | OR (95%CI) | P value | OR (95%CI) | P value |
| For AKI |  |  |  |  |
| Unadjusted | 2.66 (1.82-5.70) | <0.001 | 5.9 (2.5, 18.1) | **<0.001** |
| Adjusted for model I | 2.84 (1.90-6.06) | <0.001 | 6.3 (2.5, 20.7) | **<0.001** |
| Adjusted for model II | 4.20 (2.51-7.96) | <0.001 | 8.7 (2.3, 37.8) | **0.003** |
| Adjusted for model III | 5.45 (3.37-10.35) | <0.001 | 9.9 (2.8, 45.1) | **0.014** |
| For new AKI^1^ |  |  |  |  |
| Unadjusted | 3.09 (1.71-5.59) | <0.001 | - | - |
| Adjusted for model I | 3.23 (1.76-5.91) | <0.001 | - | - |
| Adjusted for model II | 3.28 (1.72-6.26) | <0.001 | - | - |
| Adjusted for model III | 2.49 (1.21-5.13) | 0.014 | - | - |
| For persistent AKI^1^ |  |  |  |  |
| Unadjusted | 2.82 (1.13-7.04) | 0.027 | - | - |
| Adjusted for model I | 3.05 (1.10-8.46) | 0.032 | - | - |
| Adjusted for model II | 3.19 (0.82-12.43) | 0.094 | - | - |
| Adjusted for model III | 2.89 (0.35-23.48) | 0.321 | - | - |
| For AKI progression^1^ |  |  |  |  |
| Unadjusted | 2.50 (1.01-6.16) | 0.047 | - | - |
| Adjusted for model I | 2.37 (1.05-6.64) | 0.039 | - | - |
| Adjusted for model II | 4.03 (1.12-14.51) | 0.033 | - | - |
| Adjusted for model III | 4.36 (0.69-21.71) | 0.109 | - | - |

AKI, acute kidney injury, OR, odds ratio, 95%CI, 95% confidence index, Model I adjusted for age, gender, weight, ethnicity. Model II adjusted for model I plus comorbidities and Charlson comorbidity index, score system, interventions, and drug usage. Model III adjusted for model II plus vital signs and laboratory results except for white blood count.

**Supplemental Table 4 Univariate and multivariate Cox regression analysis for clinical outcomes in validation sets**

| Methods | Internal validation set | | External validation set | |
| --- | --- | --- | --- | --- |
|  | HR (95%CI) | P value | HR (95%CI) | P value |
| For ICU mortality |  |  |  |  |
| Unadjusted | 2.03 (1.01-4.11) | 0.049 | 6.6 (2.0, 21.9) | 0.002 |
| Adjusted for model I | 2.34 (1.14-4.84) | 0.021 | 5.6 (1.6-19.3) | 0.006 |
| Adjusted for model II | 2.49 (1.07-5.77) | 0.034 | 4.9 (0.8-31.0) | 0.091 |
| Adjusted for model III | 4.07 (1.35-12.29) | 0.013 | 5.8 (0.6-43.5) | 0.358 |
| For in-hospital mortality |  |  |  |  |
| Unadjusted | 2.10 (1.39-3.16) | <0.001 | 4.7 (1.8-12.3) | 0.002 |
| Adjusted for model I | 2.18 (1.44-3.32) | <0.001 | 4.3 (1.6-11.6) | 0.004 |
| Adjusted for model II | 1.92 (1.2502.95) | 0.003 | 3.2 (0.7-15.1) | 0.144 |
| Adjusted for model III | 2.48 (1.50-4.10) | <0.001 | 6.6 (0.6-32.6) | 0.237 |

AKI, acute kidney injury, HR, hazard ratio, 95%CI, 95% confidence index, Model I adjusted for age, gender, weight, ethnicity. Model II adjusted for model I plus comorbidities and Charlson comorbidity index, HAS-BLED score, score system, interventions, and drug usage. Model III adjusted for model II plus vital signs and laboratory results except for white blood count.





**Supplemental Figure 1** The waterfall plots of the high-risk group and low risk group for the prediction of AKI (**A**), new AKI (**B**), AKI progression (**C**), and persistent AKI (**D**) for patients in the internal validation set.


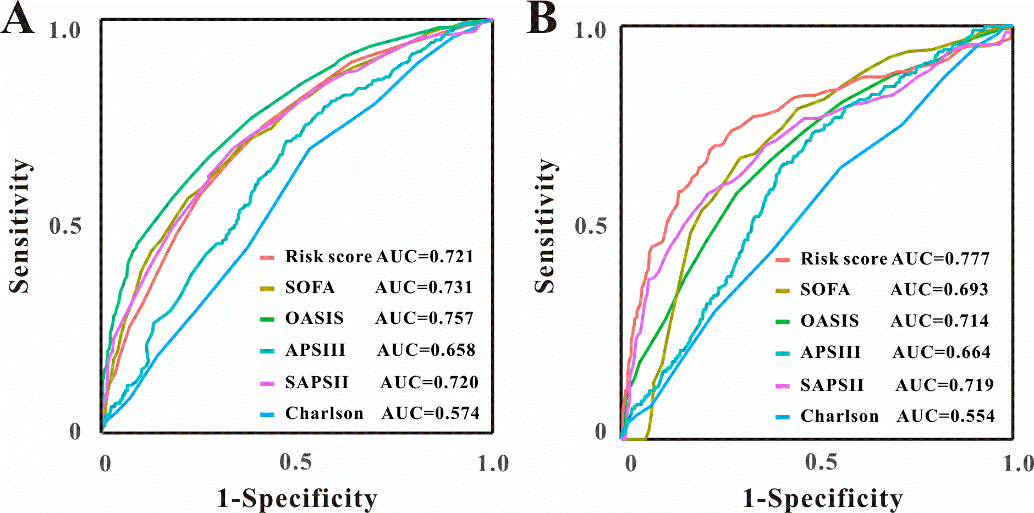


**Supplemental Figure 2.** ROC curves of risk score and severity score for the prediction of **(A)** ICU mortality and **(B)** in-hospital mortality among critically ill HCC patients.

**

**

**Supplemental Figure 3** The waterfall plots and forest plots of the risk score in individuals with HCC for ICU mortality and in-hospital mortality in the training set.





**Supplemental Figure 4** The risk score was established to detect the ICU and in-hospital mortality of patients with HCC in the internal validation set. All patients were distinguished into high and low risk based on the risk score (**A**), the relationship between survival time and prognosis of patients in the two corresponding groups (**B**), and the heatmap of inflammatory marks between the two groups (**C**). The Kaplan-Meier curves showing the in-hospital mortality (**D**) and ICU mortality (**E**) of groups with different risk. The risk score was established to detect the ICU mortality of patients with liver cancer in the internal validation set. All patients were distinguished into high and low risk based on the risk score (**F**), the relationship between survival time and prognosis of patients in the two corresponding groups (**G**), and the heatmap of inflammatory marks between the two groups (**H**).





**Supplemental Figure 5** The waterfall plots and the Kaplan-Meier curves of the risk score in individuals with HCC for clinical outcomes in the external validation set. The waterfall plots of the high-risk group and low risk group for the prediction of AKI (**A**), ICU mortality (**B**), and in-hospital (**C**) for patients in the external validation set. The Kaplan-Meier curves showing the ICU mortality (**D**) and in-hospital mortality (**E**) of groups with different risk.
